# Clinical Characteristics and Treatment Management of Seronegative Myasthenia Gravis: A Systematic Review of the Literature

**DOI:** 10.1002/mus.70130

**Published:** 2026-07-01

**Authors:** Massimiliano Ugo Verza, Mattia Schiavolin, Francesca Beretta, Sara Cornacchini, Antonio Lotti, Gregorio Spagni, Valentina Damato

**Affiliations:** ^1^ NEUROFARBA Department University of Florence Florence Italy; ^2^ Department of Neurologia D'Urgenza Azienda Ospedaliero Universitaria Careggi Florence Italy

**Keywords:** diagnosis, immunotherapy, myasthenia gravis, outcome, seronegative

## Abstract

Seronegative myasthenia gravis (SNMG) is characterized by the absence of detectable autoantibodies against known MG targets, despite clinical and electrophysiological evidence of a postsynaptic neuromuscular junction disorder. The aim of this systematic review was to better define clinical features, diagnostic clues and treatment outcome of SNMG patients. We conducted a systematic review following PRISMA guidelines, analyzing studies published from 2001 to August 2024 through MEDLINE, Embase, and Cochrane databases. A total of 31 studies (712 patients) with SNMG diagnoses based on clinical features, electrophysiology, pharmacological testing, or treatment response were included. The cohort was predominantly female (473/712, 67%) with a mean age at onset of 42 years. Isolated ocular symptoms were present in 281/500 (56%) patients, with progression to generalized MG in 113/262 (43%) within 2 years from onset. Regarding diagnostic strategies, some studies combined clinical findings consistent with MG and both electrophysiology and pharmacological testing, while others applied a less stringent diagnostic approach, leading to variability in case definitions. Moreover, only 213/448 (48%) patients underwent cell‐based assays to confirm the seronegative results after antibody screening, raising concerns of misclassification. Immunotherapy was administered to 271/333 (81%) patients, most commonly corticosteroids combined with immunosuppressants (173/271, 74%). Despite treatment, only 153/394 (39%) achieved the minimal manifestations status, and 52/220 (24%) were classified as refractory. In conclusion, SNMG is a heterogeneous condition with significant diagnostic and therapeutic challenges. Standardized diagnostic criteria, including the use of highly sensitive antibody assays, are needed to improve immunological stratification and optimize therapeutic management.

**Abbreviations**
AChE‐ I: Acetylcholinesterase inhibitor
AChR: Acetylcholine receptor
CBA: Cell‐based assay
CNIs: calcineurin Inhibitors
dSNMG: Double seronegative Myasthenia Gravis
EDX: electrodiagnostic
EFG: Efgartigimod
ELISA: enzyme linked immunosorbent assay
gMG: generalized Myasthenia Gravis
GMS: genetic myasthenic syndromes
I: improved
LRP4: low‐density lipoprotein receptor‐related protein 4
MG: Myasthenia Gravis
MGFA: Myasthenia Gravis Foundation of America
MM: minimal manifestation
MuSK: muscle‐specific kinase
NSIST: non‐steroidal immunosuppressive therapy
PIS: post interventional status
RIA: Radioimmunoassay
RNS: Repetitive Nerve Stimulation
RTX: Rituximab
SFEMG: Single‐Fiber Electromyography
SNMG: Seronegative Myasthenia Gravis
tSNMG: Triple seronegative Myasthenia Gravis

## 1 Introduction

Seronegative myasthenia gravis (SNMG) is defined by the lack of detectable autoantibodies against known MG targets, in patients otherwise exhibiting typical clinical and electrophysiological features of a post‐synaptic neuromuscular junction disorder. Approximately 10%–15% of MG cases fall into this category [1, 2]. MG is defined as double‐seronegative (dSNMG) when antibodies against neither acetylcholine receptor (AChR) nor muscle‐specific kinase (MuSK) are detected, and as triple‐seronegative (tSNMG) when antibodies against low‐density lipoprotein receptor‐related protein 4 (LRP4) are also tested and found to be negative. Importantly, the absence of detectable antibodies is influenced by the sensitivity of the antibody assay used, as some low‐affinity or conformationally specific antibodies may only be identified using advanced techniques like live cell‐based assays (CBA) [3]. The lack of antibody diagnostic confirmation makes SNMG a diagnostic and therapeutic challenge, which may result in significant diagnostic delays and possibly worse outcome. In addition, a thorough evaluation of possible alternative diagnoses should be carefully performed as the risk of misdiagnosis is higher in seronegative than in antibody‐positive MG, which may expose patients to unnecessary therapies and adverse treatment‐related events [4]. Supportive diagnostic tools such as electrodiagnostic (EDX) testing, particularly single‐fiber electromyography (SFEMG), pharmacological testing, and the ice‐pack test, may aid in the diagnosis [5], but their use varies across different centres, depending on the local availability and the operator's expertise, which can impact their diagnostic performances. Indeed, the reported sensitivity and specificity of some of these diagnostic tools, such as SFEMG and the ice‐pack test, vary greatly across studies [6, 7, 8]. More generally, given the absence of universally accepted diagnostic criteria for SNMG, different sets of criteria have been used in the literature to define these patients, which limits the comparability of results and the design of clinical trials. For all these reasons, SNMG remains challenging in terms of optimal diagnostic strategy, best treatment management and disease outcome.

The aim of this systematic review is to address these aspects by providing a comprehensive overview of SNMG based on published studies, with a focus on diagnostic strategies, clinical characteristics, treatment management, and disease outcomes.

## 2 Methods

### 2.1 Systematic Literature Review

#### 2.1.1 Search Strategy

We performed a systematic review according to the updated Preferred Reporting Items for Systematic Reviews and Meta‐Analyses statement guidelines (PRISMA) [9]. Three reviewers (M.U.V., M.S., and F.B.) independently searched articles published from 2001, the year in which anti‐MuSK antibodies were first identified [10], up to August 2024 using the following databases: MEDLINE, Embase, and Cochrane. The following search query was used: *(seronegative myasthenia gravis OR double‐negative myasthenia gravis OR triple‐negative myasthenia gravis OR negative antibody myasthenia gravis OR SNMG) AND (outcome OR immunotherapy)*. For studies with partial data reported, the leading authors were contacted for further information.

#### 2.1.2 Selection Criteria

The studies included in the analysis were selected based on the following criteria:

1. Original studies published in English (reviews, opinion papers, editorials, comments, conference abstracts, and animal studies were excluded) that included patients with a diagnosis of SNMG according to two core criteria and at least one ancillary criterion:
  Core criteria:
  - Clinical findings consistent with the disease,
  - Undetectable anti‐AChR and anti‐MuSK antibodies (dSNMG) OR undetectable anti‐AChR, anti‐MuSK, and anti‐LRP4 antibodies (tSNMG) tested by the best available assay in each study analyzed
    Ancillary criteria:
  - Findings consistent with MG on EDX testing
  - Response to pharmacological testing (edrophonium or neostigmine),
  - Clinical response to therapeutic anticholinesterase inhibitor drugs,
  - Clinical response to immunotherapy.
2. Availability of relevant clinical information
  Using the search parameters, 570 records were identified. After removing duplicates, 459 individual records were evaluated for eligibility based on their titles and abstracts by two independent reviews (MUV and MS), resulting in 110 papers. After full text appraisal by at least two reviewers (MUV, MS, and FB), 31 papers were retained for final analysis [11, 12, 13, 14, 15, 16, 17, 18, 19, 20, 21, 22, 23, 24, 25, 26, 27, 28, 29, 30, 31, 32, 33, 34, 35, 36, 37, 38, 39, 40, 41]. The flow chart of the systematic review strategy is shown in Figure S1.

#### 2.1.3 Data Extraction and Analysis

Data extraction was independently performed by three reviewers (MUV, MS, and FB) using a standardized data collection form. A double‐checking process was conducted by two independent reviewers to ensure accuracy. Any discrepant data were reviewed and resolved after re‐examination of the source studies. For each study, the following data, when available, were retrieved: article title, author, year, journal, number of cases, patient age and sex, age at onset, Myasthenia Gravis Foundation of America (MGFA) classification at disease onset, clinical characteristics, methods of diagnosis, EDX results, thymus pathology, antibody tested and assay used, treatment, response to therapy, and clinical outcome according to MGFA‐post intervention status (MGFA‐PIS) [42].

#### 2.1.4 Statistical Analysis

The collected data were combined to provide information on demographics, clinical characteristics, diagnostic methods, and treatment approaches, described using means (calculated as weighted averages, with weights proportional to the sample size of each included study), frequencies, and proportions. Where applicable, between‐group comparisons were conducted using Fisher's exact test or the Chi‐square test for categorical data. Statistical analysis was performed using Prism version 10.5.0 (GraphPad, San Diego, CA).

## 3 Results

### 3.1 Clinical and Demographic Features of SNMG

The 31 included studies [11, 12, 13, 14, 15, 16, 17, 18, 19, 20, 21, 22, 23, 24, 25, 26, 27, 28, 29, 30, 31, 32, 33, 34, 35, 36, 37, 38, 39, 40, 41] comprised a total of 712 patients diagnosed with SNMG. The demographic and clinical data of the overall cohort are summarized in Tables 1 and S1.

**TABLE 1:** Demographic and clinical characteristics of patients included in the systematic review.

Studies, *n*	SNMG patients, *n*/Sex, F:M	Age at onset, years, mean (range)	Ocular at onset, *n* (%)	Bulbar involvement, *n* (%)	MGFA class ≥ III during disease course, *n*	Thymectomy *n* (%)/(Thymoma), *n* (%)
31	712/473:239	42 (2–83)	281/500 (56)	231/569 (41)	88/500 (18)	8/425 (2)/1/425 (< 1)

Abbreviations: MGFA, myasthenia gravis foundation of America; SNMG, seronegative myasthenia gravis.

**FIGURE 1:**
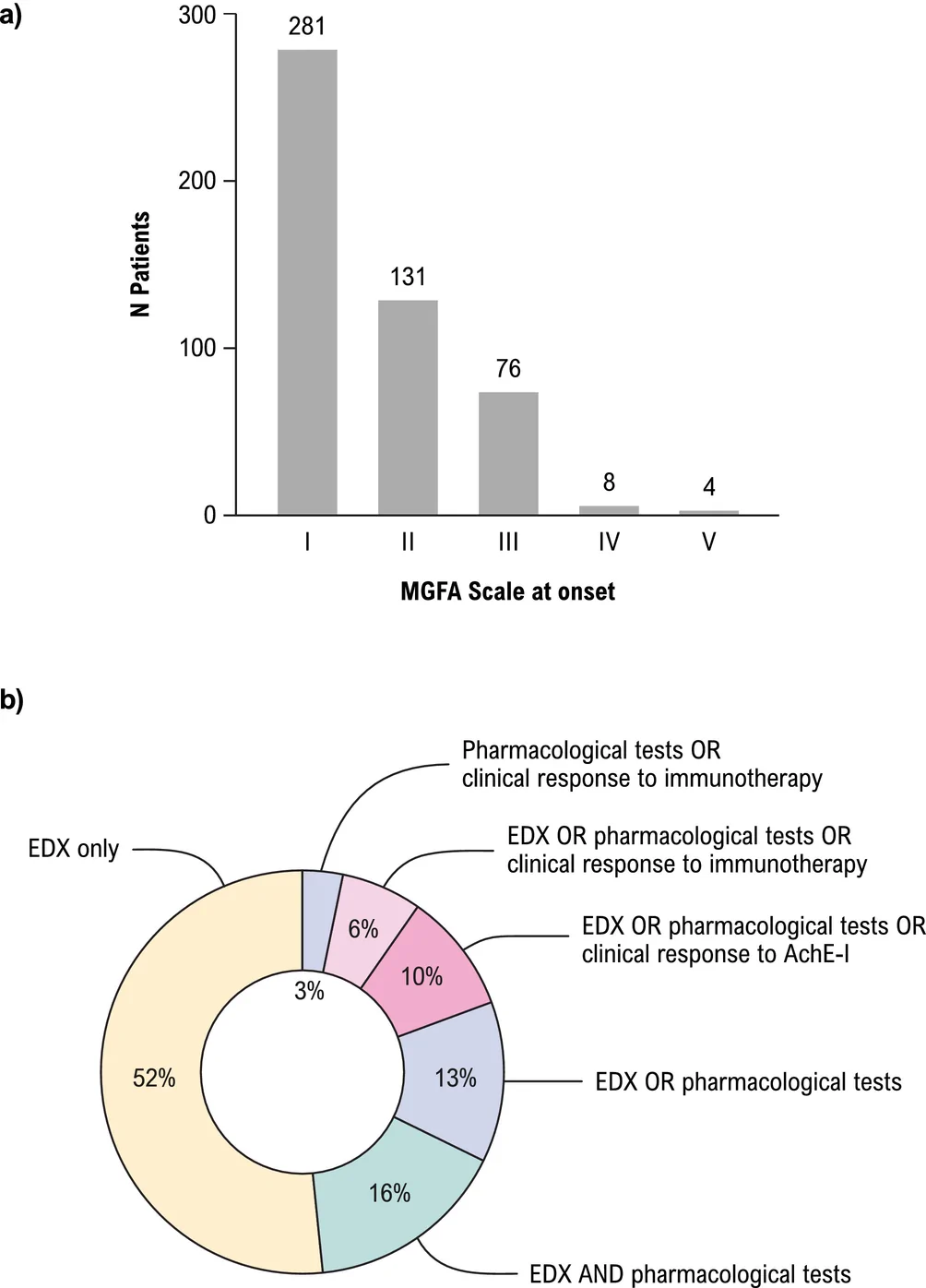
Clinical features and diagnostic criteria used for the diagnosis of SNMG. Distribution based on disease severity at onset according to MGFA classification in the population included in the systematic review (a). Proportion of patients diagnosed using the ancillary criteria for the diagnosis of SNMG by the studies included in the systematic review. All studies fulfilled the two core diagnostic criteria: (1) clinical findings consistent with myasthenia gravis and (2) negative serological testing for anti‐AChR and anti‐MuSK antibodies. EDX testing includes either RNS or SFEMG. Diagnostic pharmacological tests include the edrophonium test or the neostigmine test (b). AChE‐I, Acetylcholinesterase inhibitor; AChR, Acetylcholine receptor; EDX Electrodiagnostic; MGFA, Myasthenia Gravis Foundation of America; MuSK, Muscle‐specific kinase; RNS, Repetitive Nerve Stimulation; SFEMG, Single‐Fiber Electromyography; SNMG, Seronegative Myasthenia Gravis.

MGFA classification at onset was available for 70% of patients. Of these, purely ocular symptoms at onset were observed in most patients, accounting for 56%, while among patients with generalized symptoms, 26% had mild disease (MGFA Class II), and 18% had moderate to severe disease at onset (MGFA Class III or higher) (Figure 1a). Follow‐up data on the clinical course were available for 262/281 (93%) patients who initially presented with ocular symptoms, showing that 113/262 patients (43%) progressed to generalized myasthenia within the first 2 years of disease. Bulbar involvement during the disease course was reported in 231/569 patients (41%).

A total of 102 out of 425 patients (24%) underwent thymectomy; among them, 8/425 patients (2%) had thymoma, and 1/425 (< 1%) had thymic carcinoma.

### 3.2 Diagnosis of SNMG

#### 3.2.1 Diagnostic Criteria for SNMG

Among the 31 studies included in this review, we observed notable heterogeneity in the applied diagnostic criteria. In 16/31 studies (52%) the diagnosis was established based on the core criteria (see Methods section) combined with the presence of positive electrophysiological tests (repetitive nerve stimulation (RNS) or SFEMG). Five/31 studies (16%) employed more stringent diagnostic criteria, requiring the core criteria plus both positive electrophysiological findings and a positive pharmacological test. In contrast, 10/31 studies (32%) adopted a more permissive approach, using the core criteria along with at least one additional criterion among EDX testing, pharmacological testing, response to pyridostigmine, or immunotherapy (Figure 1b). A detailed description of the criteria used in each study is provided in Table S2. This variability underscores the absence of standardized diagnostic pathways for SNMG and highlights the potential impact on patient selection, study populations, and reported outcomes.

#### 3.2.2 Type of Antibody Testing Performed

Data regarding the type of assay used for antibody detection were available in 448/712 (63%) patients. All sera were tested using radioimmunoassay (RIA) for anti‐AChR and anti‐MuSK antibodies, while a second step evaluation with AChR and MuSK CBAs was done in 213/448 (48%) patients (confirming the negativity in all cases, as per inclusion criteria). A temporal analysis of the implementation of CBA as a confirmatory diagnostic approach indicated a limited adoption during the first decade following its introduction (2008–2018), with only 2 out of 7 studies (29%) reporting its use. In the subsequent period (2018–2024), the proportion of studies employing CBA increased to 5 out of 11 (45%), suggesting a growing recognition of its diagnostic value and a progressive shift in clinical and research practices.

Only 288/612 (47%) patients (total of patients in our review after 2011, year in which anti‐LRP4 antibodies were firstly described [43]) were tested for anti‐LRP4 antibodies in addition to anti‐AChR and anti‐MuSK antibodies. Of these, 123/288 (43%) were tested using CBA, 11/288 (4%) were tested using luciferase immunoprecipitation system, and 154/288 (53%) patients were tested using enzyme linked immunosorbent assay (ELISA).

#### 3.2.3 Electrodiagnostic Testing

Electrodiagnostic testing was performed in 557/712 (78%) patients. However, detailed information regarding the specific type of test performed was available in only 255/557 (46%) of them. Most patients (248/255, 97%) underwent RNS, which yielded positive results in 143/248 cases (58%). When considering only patients with generalized MG, RNS was positive in 73/91 (80%) cases. This finding underscores the reduced sensitivity of RNS in patients with purely ocular forms of MG, which comprise the majority of SNMG patients.

SFEMG results were reported in 144/712 (20%) patients. The examination was performed as second line testing after RNS in 137/144 (95%) of them, whereas 7 patients had SFEMG as the only performed test. SFEMG was positive in 137/144 (95%) SNMG patients.

### 3.3 Treatment of SNMG

**FIGURE 2:**
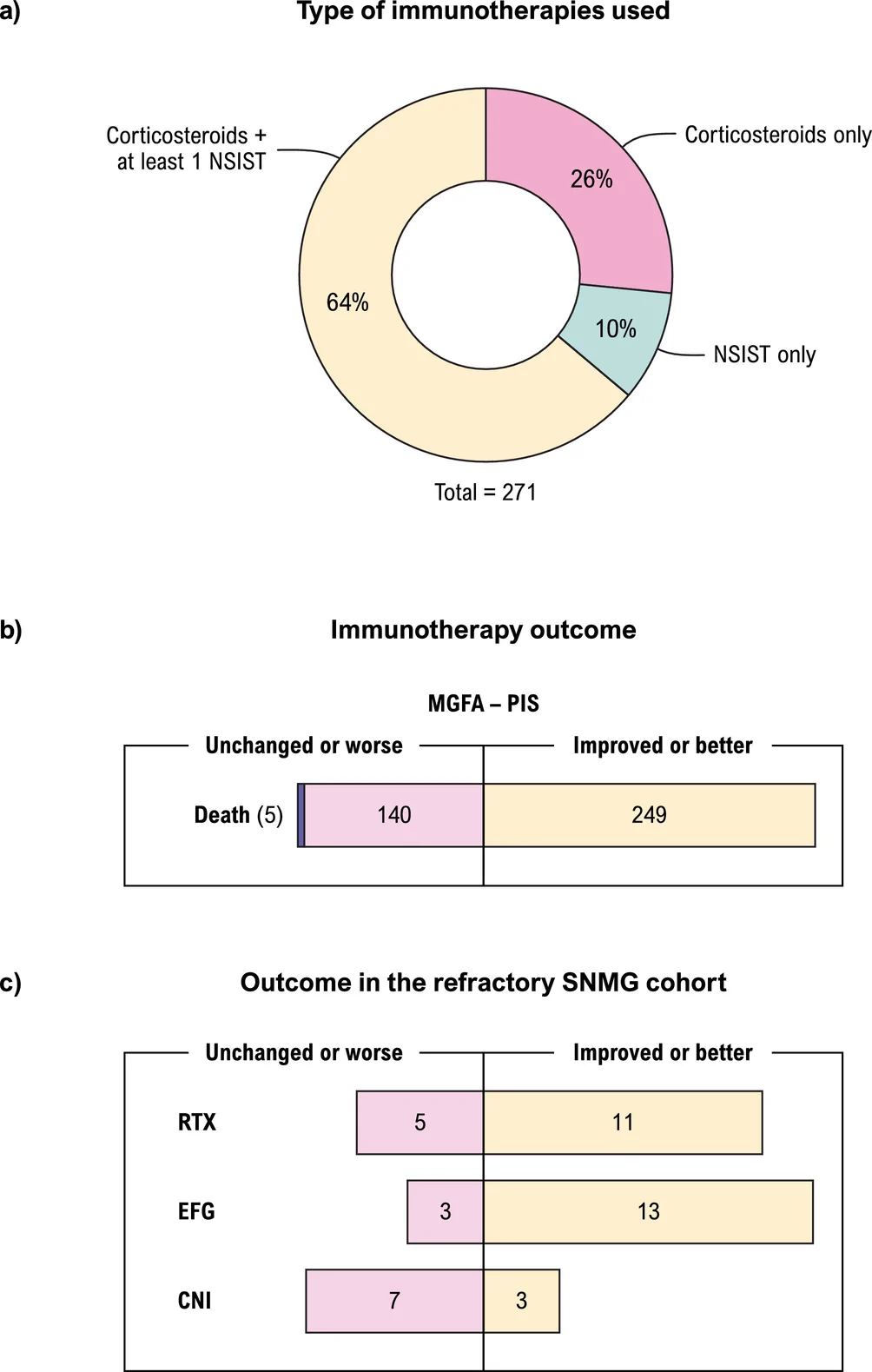
Immunotherapy and outcome of SNMG patient. Proportion of patients undergoing different immunotherapy drugs (a). Proportion of outcome of SNMG patients based on response to pharmacological therapies according to MGFA‐PIS (b). Outcome of treatment‐refractory SNMG patients according to three different types of immunotherapies (*n* = 16 Rituximab; *n* = 16 Efgartigimod; *n* = 10 Calcineurin Inhibitors). Favorable outcome was defined as “Improved or Better” according to MGFA—PIS (c). CNI, calcineurin; EFG, Efgartigimod; MGFA—PIS, Myasthenia Gravis Foundation of America—Post Intervention Status; NSIST, non‐steroidal immunosuppressive therapy; RTX, rituximab; SNMG, Seronegative Myasthenia Gravis.

Treatment data were available for 333/712 (47%) patients. A total of 53/333 patients (16%) received symptomatic therapy alone, whereas most patients (271/333 patients, 81%) underwent immunotherapy. Of this latter group, 72 were treated with corticosteroids alone, 26 received only non‐steroidal immunosuppressive therapy (NSIST), and 173 were treated with a combination of corticosteroids and NSIST (Figure 2a). The most prescribed NSIST drug was azathioprine (74 patients, 27%), followed by mycophenolate mofetil (32 patients, 12%). Among biological therapies, rituximab was the most used (32 patients, 12%), followed by efgartigimod (22 patients, 8%).

### 3.4 Outcome of SNMG

We collected data on clinical outcomes from 394/712 patients in which the MGFA‐PIS was specified. Patients were grouped based on their clinical response into two categories: those who achieved a favorable outcome, defined as an MGFA‐PIS of Minimal Manifestations or better (MM‐or‐better), and those who did not reach this threshold. Among the included patients, 153/394 (39%) achieved a favorable outcome. When considering a broader definition of clinical response, defined as a MGFA‐PIS of Improved or better (I‐or‐better), 63% were found to meet this criterion. Importantly, 35% had a MGFA‐PIS “unchanged or worse” and 1% died of MG (Figure 2b).

#### 3.4.1 Refractory Patients

Among 220/712 (31%) patients for whom follow‐up and treatment data were available to assess refractoriness, 52/220 (24%) met the criteria for refractory myasthenia gravis (MG) [44, 45]. Of these, 43/52 (83%) patients were tested for anti‐AChR and anti‐MuSK antibodies using RIA, but the seronegative status was confirmed by live CBA in only 6/43 (14%) of them. In 9/52 (17%) patients, data regarding the type of test performed was not specified. Anti‐LRP4 antibodies were tested in 10/52 (19%) patients. All patients were reported to have electromyographic findings consistent with MG, all showing an abnormal response either by RNS and/or SFEMG.

We conducted a comparative analysis between refractory and non‐refractory patients, and we observed an association between bulbar symptoms and poor therapeutic response, whereas ocular onset showed a negative association with refractoriness (Table 2). Second/third line treatment after steroids and at least one NSIST, details were available in 44/52 refractory SNMG patients: 39% were treated with rituximab, 36% with efgartigimod, 23% with calcineurin inhibitors, and 2% with eculizumab. Of these, 27% achieved a favorable outcome, whereas 64% demonstrated at least some improvements (MGFA‐PIS of I‐or‐better). 34% did not respond to these therapies; one patient died due to MG‐related complications. Outcomes related to therapies are shown in Figure 2c.

**TABLE 2:** Comparison between therapy responsive and therapy refractory patients.

	Refractory	Non refractory	*p*
Patients, *n* (%)	52/220 (24)	168/220 (76)	
Female, *n* (%)	39/52 (75)	109/168 (65)	0.2
Age at onset, years: mean (range)	36.4 (27–75)	36.7 (2–83)	
MGFA class at onset available, *n* (%)	31/52 (60)	87/168 (52)	
I	0/52 (0)	15 (17.2)	0.01
II	12/52 (39)	35/168 (40)	1
III	17/52 (54)	31/168 (35)	0.08
IV	2/52 (6)	3/168 (3)	0.6
V	0/52 (0)	3/168 (3)	0.5
Moderate to severe disease, *n* (%)	19/52 (61)	37/168 (43)	0.09
Data on bulbar symptoms available, *n* (%)	36/52 (70)	146/168 (87)	
Bulbar involvement, *n* (%)	25/36 (69)	63/146 (43)	0.005
Thymoma, *n* (%)	0/52 (0)	2/168 (1)	1

Abbreviation: MGFA, myasthenia gravis foundation of America.

## 4 Discussion

This systematic review highlights the substantial diagnostic and clinical heterogeneity among published studies on SNMG. The absence of standardized diagnostic criteria, inconsistent application of EDX testing, and variable use of high‐sensitivity antibody assays collectively contribute to uncertainty in disease classification. As a result, misdiagnosis remains a major concern: some patients labeled as SNMG may in fact have other neuromuscular junction disorders, genetic myasthenic syndromes (GMS), or unrelated neuromuscular diseases. Only 78% of patients in the included studies underwent EDX confirmation, and in several reports, diagnosis relied mainly on clinical presentation and therapeutic response. The absence of EDX testing in approximately one fifth of cases represents a major limitation, as it precludes confirmation of a neuromuscular junction transmission defect and increases the risk of including non‐MG cases. It is important to note that neuromuscular junction instability is not exclusive to autoimmune MG. Conditions characterized by active denervation and reinnervation, such as Guillain–Barré syndrome, motor neuron disease (ALS), and inflammatory myopathies, may also show increased neuromuscular jitter and blocking on SFEMG, potentially leading to false‐positive interpretations. In some of these disorders, minimal or transient symptomatic improvement with acetylcholinesterase inhibitors has also been reported, further complicating the diagnostic picture. For this reason, an adequate EDX evaluation for suspected SNMG should not be limited to RNS or SFEMG alone but must include a comprehensive EDX study, with conventional needle EMG and nerve conduction studies to exclude neurogenic or myopathic processes before confirming a diagnosis of seronegative MG.

Furthermore, criteria for “clinical therapeutic response” were inconsistently defined and may have inflated diagnostic confidence, given that placebo response rates in MG trials can exceed 30%–40% [46]. Equally concerning is the incomplete use of advanced antibody testing. Only a minority of studies employed live cell‐based assays (CBAs) for anti‐AChR or anti‐MuSK antibodies, and anti‐LRP4 antibody testing was performed in fewer than half of the cases, often with suboptimal methods. Consequently, some patients may have been misclassified as “seronegative” due to limited assay sensitivity rather than true antibody negativity. The increasing adoption of live CBAs in recent years is encouraging and will likely improve immunological stratification in future research. Importantly, none of the studies systematically incorporated genetic testing into their diagnostic workflow, representing a significant gap in current practice. This omission is particularly relevant because GMS and other inherited disorders can closely mimic autoimmune MG, especially in antibody‐negative patients. Recent data indicate that up to 14% of patients clinically diagnosed with SNMG harbor pathogenic variants consistent with GMS [47]. Incorporating targeted or panel‐based genetic screening into the diagnostic pathway, particularly for early‐onset, atypical, or treatment‐refractory cases, could therefore prevent misclassification and improve patient management.

The demographic and clinical profile emerging from this review aligns with previous observations of a predominance of early‐onset disease and female sex [18]. Over half of the patients presented with purely ocular symptoms at onset, and only one‐third progressed to generalized disease within 2 years. These findings reinforce the established notion that seronegative status is more common in early‐onset and ocular MG, whereas late‐onset forms are more frequently associated with anti‐AChR seropositivity [48].

Contrary to earlier assumptions that SNMG represents a mild or self‐limiting subtype [37], over 30% of patients experienced moderate to severe disease during their clinical course. The widespread use of immunotherapy, reported in more than 80% of cases, highlights both the perceived severity of disease and the potential for overtreatment in diagnostically uncertain cases. Despite extensive immunosuppression, fewer than 40% of patients achieved minimal manifestation‐or‐better status, and over one‐third remained unchanged or worsened. These outcomes are markedly lower than those reported in seropositive MG cohorts, such as the Duke Myasthenia Gravis Registry [49], where remission rates approach 70%. Both diagnostic uncertainty and suboptimal therapeutic targeting likely contribute to this discrepancy. Approximately one in four patients met criteria for refractory MG, a proportion exceeding that reported in AChR‐positive disease (10%–15%) [50]. Within this subgroup, biologic therapies such as rituximab and efgartigimod showed variable but notable responses [14, 36, 38, 39], suggesting that a subset of SNMG patients harbor autoimmune mechanisms amenable to targeted immunomodulation. However, incomplete antibody testing in many refractory cases raises the possibility of undetected antibodies or novel antigenic targets. Recent immunophenotyping studies revealing altered B‐cell subsets and immune dysregulation in SNMG [51] support this hypothesis, emphasizing the need for biological stratification within this heterogeneous spectrum.

Several limitations of this review must be acknowledged. Many included studies were retrospective and lacked consistent diagnostic criteria, limiting comparability across cohorts. The inconsistent use of high‐sensitivity antibody assays and electrophysiological confirmation may have led to misclassification. Outcome data were incompletely reported, and therapeutic response definitions varied widely. Finally, the lack of prospective, controlled studies in SNMG restricts conclusions about treatment efficacy and disease trajectory.

Taken together, these findings underscore the need for standardized diagnostic algorithms for SNMG, as the one recently proposed by the 275th ENMC workshop on SNMG [52]. Such frameworks should mandate EDX confirmation, comprehensive antibody testing with the most sensitive available methods, and reasonable exclusion of alternative diagnoses, including GMS and other neuromuscular disorders, through genetic or targeted laboratory investigations. Given the decreasing cost of genetic sequencing, incorporating extended neuromuscular gene panels could become both feasible and cost‐effective.

In conclusion, SNMG represents a diagnostically and therapeutically heterogeneous group of patients. Future studies should aim to refine classification by integrating high‐sensitivity serological, electrophysiological, and genetic data, supported by prospective validation. From a clinical perspective, while adequate symptom control remains essential, caution must be exercised to avoid unnecessary immunosuppression in unconfirmed cases. Finally, given that a subset of SNMG patients exhibit immunopathogenic features and respond to biologics, future therapeutic developments should explicitly include antibody‐negative populations rather than restricting access to antibody‐positive cases.

## Author Contributions

**Massimiliano Ugo Verza:** data curation, formal analysis, writing – original draft and editing. **Mattia Schiavolin:** data curation, formal analysis, writing – original draft and editing. **Francesca Beretta:** data curation, formal analysis, writing – review and editing. **Sara Cornacchini:** data curation, writing – review and editing. **Antonio Lotti:** writing – review and editing. **Gregorio Spagni:** supervision, validation, writing – review and editing. **Valentina Damato:** conceptualization, methodology, supervision, validation, writing – original draft, review and editing.

## Funding

This work was supported by a grant from the EJP‐RARE DISEASE 2023 project ‘OptiMyG’.

## Ethics Statement

We confirm that we have read the Journal's position on issues involved in ethical publication and affirm that this report is consistent with those guidelines.

## Conflicts of Interest

V.D. has received public speaking honoraria and compensation for advisory boards and/or consultation fees from UCB, Alexion, Dianthus, Roche. G.S. received public speaking/consulting honoraria from Alexion, AMGEN, and Argenx. The remaining authors declare no conflicts of interest.

## Supporting information


**Table S1:** Clinical and demographic data of the overall cohort.
**Table S2:** Diagnostic criteria.
**Figure S1:** Preferred Reporting Items for Systematic Reviews and Meta‐Analyses (PRISMA) flow diagram showing systematic review selection process.

## Data Availability

The data that support the findings of this study are available from the corresponding author upon reasonable request.
