# Supplementary material for: Clinical Characteristics and Treatment Management of Seronegative Myasthenia Gravis: A Systematic Review of the Literature
Source: Muscle Nerve. 2026 Jul 1;74(Suppl 1):S76–83. doi: 10.1002/mus.70130 (PMC13599449; doi:10.1002/mus.70130)
Supplement: Supplementary file 1 [file MUS-74-S76-s001.pdf]

Supplementary figure 1

Preferred Reporting Items for Systematic Reviews and Meta-Analyses (PRISMA) flow diagram showing systematic review selection process

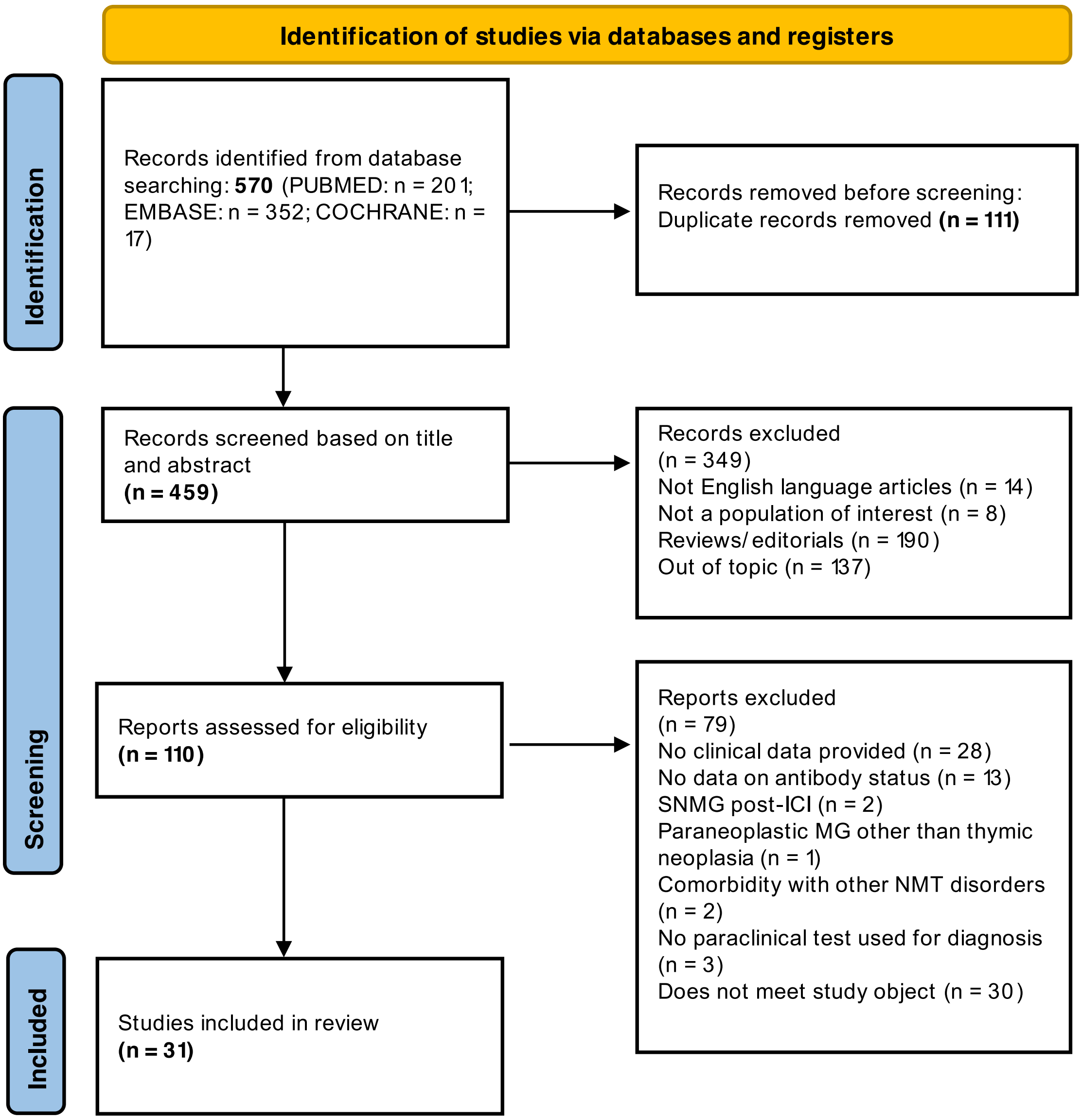

Supplementary Table 1, clinical and demographic data of the overall cohort

Authors, year	Year	Country	Type of study	SNMG patients, n	Sex, F (n)	Age at Onset, mean	Ocular at onset, n	Bulbar (at any time), n	MGFA ≥ III during disease course, n	Thymectomy, (Thymoma), n
Pasqualin et al	2020	Italy	Retrospective cohort study	21	12	44,8	10	na	7	5
Afanasiev et al	2017	France	Case series	4	3	na	na	4	3	2
Horiuchi et al	2024	Japan	Case series	16	13	47,1	0	5	7	1
Romi et al	2005	Norway	Retrospective cohort study	17	11	36	1	na	na	3
Roda et al	2018	US	Historical case series	4	na	na	0	4	4	2
Lebrun et al	2008	France	Case series	2	2	na	na	na	na	1
Azajnac et al	2014	Serbia	Case report	1	0	22	0	1	1	1
Rigamonti et al	2011	Italy	Case report	1	0	60	0	1	1	1 (1)
Shin J et al	2012	UK	Prospective	59	42	na	14	24	10	13 (3)
Dan et al	2014	Switzerland	Case report	1	1	42	0	1	1	0
Yeo et al	2018	US	Case report	1	1	79	0	1	1	0
Gol et al	2022	Turkey	Retrospective cohort study	1	1	34	0	1	1	0
Dos Santos et al	2020	France	Retrospective cohort study	4	4	21,25	0	na	2	2
Frangiamore et al	2023	Italy	Case report	1	1	36	1	1	1	1
Frangiamore et al	2024	Italy	Prospective cohort study	5	5	42,2	0	2	4	3 (1)
Ruetsch-Chelli et al	2021	France	Case series	4	3	45,75	na	na	na	4
Samal et al	2020	India	Retrospective cohort study	9	4	20	2	2	7	1
Zhao et al	2024	China	Case series	2	2	41,5	0	2	2	0
Tozawa et al	2017	Japan	Case report	1	0	3	0	1	1	0
Ziadini et al	2022	Iran	Retrospective cohort study	8	5	39	na	na	na	na
Veltsista et al	2022	Greece	Retrospective cohort study	6	4	38,8	na	na	5	5
Ware et al	2012	Australia	Case series	1	na	6	na	na	0	0
Okuzono et al	2024	Japan	Cross-sectional study	10	10	31,8	na	10	10	1
Huda et al	2016	South Africa	Retrospective cohort study	36	32	47,8	0	34	21	na
Martinez-Harms et al	2024	Canada	Retrospective cohort study	80	52	57,5	37	na	13	2 (2)
Huang et al	2007	Taiwan	Retrospective cohort study	39	28	34,6	19	21	7	15
Evoli et al	2003	Italy	Retrospective cohort study	41	21	39	0	24	16	22
Rodriguez Cruz et al	2015	UK	Retrospective cohort study	26	22	38	11	12	9	5 (1)

Damato et al	2022	Italy	Retrospective cohort study	59	39	na	na	18	17	12
Galassi et al	2018	Italy	Prospective cohort study	98	51	55,2	98	0	0	na
Rivner et al	2020	US	Retrospective cohort study	154	104	46,75	88	62	60	na

SNMG, seronegative myasthenia gravis, MGFA, myasthenia gravis foundation of America.

Supplementary table 2, Diagnostic criteria

**Authors, year**	**Country**	**Type of study**	**N of SNMG patients**	**Core criteria**	**Ancillary criteria 1b.RNS+ or SFEGM+; 2b. Pharmacological test +; 3b. clinical improve with AChEIs; 4b. Response to IT**					
					**1b**	**1b or 2b**	**1b and 2b**	**1b or 2b or 3b**	**1b or 2b or 4b**	**2b and 4b**
Pasqualin et al, 2020	Italy	Retrospective cohort study	21	X		X				
Afanasiev et al, 2017	France	Case series	4	X		X				
Horiuchi et al, 2024	Japan	Case series	16	X		X				
Romi et al, 2005	Norway	Retrospective cohort study	17	X				X		
Roda et al, 2018	US	Historical case series	4	X	X					
Lebrun et al, 2008	France	Case series	2	X			X			
Azajnac et al, 2014	Serbia	Case report	1	X			X			
Rigamoni et al, 2011	Italy	Case report	1	X	X					
Shin J et al, 2012	UK	Prospective	59	X	X					
Dan et al, 2014	Switzerland	Case report	1	X	X					
Yeo et al, 2018	US	Case report	1	X	X					
Gol et al, 2022	Turkey	Retrospective cohort study	1	X	X					
Dos Santos et al, 2020	France	Retrospective cohort study	4	X	X					
Frangiamore et al, 2023	Italy	Case report	1	X				X		
Frangiamore et al, 2024	Italy	Prospective cohort study	5	X				X		
Ruetsch-Chelli et al, 2021	France	Case series	4	X	X					
Samal et al, 2020	India	Retrospective cohort study	9	X	X					
Zhao et al, 2024	China	Case series	2	X			X			
Tozawa et al, 2017	Japan	Case report	1	X			X			
Ziadini et al, 2022	Iran	Retrospective cohort study	8	X	X					
Veltsista et al, 2022	Greece	Retrospective cohort study	6	X	X					

Ware et al, 2012	Australia	Case series	1	X						X
Okuzono et al, 2024	Japan	Cross-sectional study	10	X	X					
Huda et al, 2016	South Africa	Retrospective cohort study	36	X					X	
Martinez-Harms et al, 2024	Canada	Retrospective cohort study	80	X	X					
Huang et al, 2007	Taiwan	Retrospective cohort study	39	X			X			
Evoli et al, 2003	Italy	Retrospective cohort study	41	X	X					
Rodriguez Cruz et al, 2015	UK	Retrospective cohort study	26	X					X	
Damato et al, 2022	Italy	Retrospective cohort study	59	X	X					
Galassi et al, 2018	Italy	Prospective cohort study	98	X		X				
Rivner et al, 2020	US	Retrospective cohort study	154	X	X					

SNMG, seronegative myasthenia gravis; RNS, repetetive nerve stimulation; SFEGM, single-fiber electromyography; AChEIs, Acetylcholinesterase Inhibitors; IT, immunotherapy.
